# *Enterobacter nimipressuralis *as a cause of pseudobacteremia

**DOI:** 10.1186/1471-2334-10-315

**Published:** 2010-10-29

**Authors:** Dong-Min Kim, Sook Jin Jang, Ganesh Prasad Neupane, Mi-Sun Jang, Se-Hoon Kwon, Seok Won Kim, Won Yong Kim

**Affiliations:** 1 Division of Infectious Diseases, Department of Internal Medicine, Chosun University, School of Medicine Gwangju City, Republic of Korea; 2 Department of Laboratory Medicine, Chosun University, School of Medicine Gwangju City, Republic of Korea; 3 Department of Neurosurgery, Chosun University, School of Medicine Gwangju City, Republic of Korea; 4 Research Center for Resistant Cells, Chosun University, School of Medicine Gwangju City, Republic of Korea; 5 Department of Public Health, Chosun University, School of Medicine, Gwangju City, Republic of Korea; 6 Department of Microbiology, Chung-Ang University, College of Medicine, Seoul, Republic of Korea

## Abstract

**Background:**

The clinical significance of the *Enterobacter nimipressuralis *as human pathogens remains unclear.

**Case presentations:**

The microbiologic culture monitoring system of sterile body fluids revealed on an episode of *Enterobacter cloacae *and *Enterobacter amnigenus *in blood culture results on the same day; the antibiotic sensitivity and MIC were nearly the same for both species. First patient was a healthy woman with postmenopausal syndrome, while second patient with herpes zoster. Both patients had febrile sensations without signs of bacteremia. *E. amnigenus *was also cultured from the unused package of salined cotton in the container through epidemiologic investigation. The cultured *Enterobacter *species were all identified as *E. nimipressuralis *through *hsp60 *gene sequencing and infrequent-restriction-site PCR (IRS-PCR).

**Conclusion:**

When an unusual microorganisms such as *E. nimipressuralis *is isolated from blood of a patient with no clinical signs of sepsis, a pseudobacteremia should be suspected. When the antibiogram and MIC test results of bacterial cultures from two or more patients are nearly the same, although the species involved may appear different, it may be necessary to prove that they are the same species through molecular methods. The microbiologic cultures monitoring system will probably help to detect pseudobacteremia and other pseudo infections through reliable and fast identification.

## Background

Chosun University Hospital, Gwang-ju, Korea, is a 650-bed, tertiary care teaching hospital. Every day in our hospital, an infectious disease specialist monitors all the results of the hospital's microbiologic cultures from sterile body fluids such as blood, cerebrospinal fluid (CSF), pleural fluid, ascites, joint fluid and localized fluid collection using a computerized monitoring system. This monitoring system revealed an episode of *Enterobacter nimipressuralis *pseudobacteremia caused by contaminated saline cotton during the preparation of blood culture samples. Here we describe two cases of *Enterobacter *species pseudobacteremia.

## Case presentation

### Case 1

A 54-year-old female patient presented with myalgia and febrile sensation to outpatient Division of Gastroenterology and Hepatology in Chosun University Hospital. She had a history of liver cirrhosis due to hepatitis B virus, and had gone surgery because of a pituitary adenoma 1 year ago. The patient did not have any limitations of daily living without major health issues after the surgery. Her physical examination revealed newly formed painful grouped vesicles on the trunk. Blood culture resulted in the growth of *Enterobacter amnigenus*. The vital signs and laboratory data did not correlate with the finding of the bacteremia. Antiviral agent for the herpes zoster was used with improvement in pain and crust formation of the vesicles. The patient was treated with ciprofloxacin according to the culture results. The follow up blood culture revealed no sign of any growth of bacteria. The patient was discharged on the 10^th ^hospital day without any complications.

### Case 2

A 41-year-old female presented with febrile sensation, hot flush, and general weakness for two months to outpatient Department of Obstetrics and Gynecology. The patient had no significant medical history prior to the visit to the hospital. Physical examination was normal, and there was no clinical signs of infection. However, blood culture resulted in the growth of *Enterobacter cloacae*. Ciprofloxacin was started according to the culture results. There was no sign of fever or systemic bacteremia, and the patient was discharged on the 5^th ^hospital day with an impression of postmenopausal syndrome.

## Discussion

When the above two patients visited outpatient clinic, the doctors who were in charge just ordered blood culture afraid of systemic infection, because they had complained of febrile sensation in spite of ambiguous clinical signs or symptoms. The microbiologic cultures monitoring system revealed that *Enterobacter *species were isolated from the blood cultures of two patients, which was performed in the outpatient blood sampling room. One species was classified as *E. cloacae *and the other *E. amnigenus*, yet the sensitivity and MIC test results were almost same. A common source of contamination or "pseudobacteremia" was suspected because the both patients had positive cultures and no clinical signs of sepsis. An epidemiologic investigation was initiated to determine the origin and cause of the *Enterobacter *species bacteremia. We reviewed clinical records of the two patients, and examined the medical procedures that they had undergone. One patient was a healthy woman, while the other was a woman with liver cirrhosis. The laboratory findings were within the normal range. No common exposures that might be related to the positive blood cultures other than venipuncture in the outpatient blood sampling room were noted. When reviewing the procedure for drawing blood and the disinfectant used, suspicion fell on the salined cotton because new supplies of salined cotton had not been delivered for the past five days. We took environmental specimens, including samples from the antiseptic solution and the salined cotton, for examination, and bacteria classified as *E. amnigenus *were cultured from the unused packages of salined cotton in the container that had been used in the blood sampling room for wiping the skin with disinfectant prior to venipuncture for blood culture.

**Figure 1:**
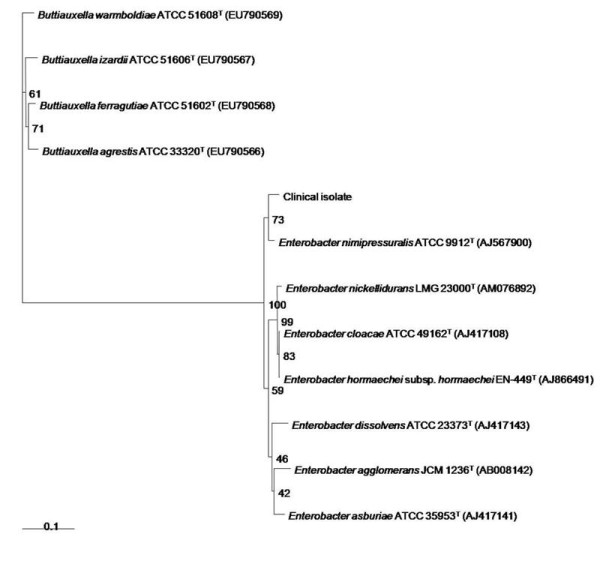
**Phylogetetic tree based on partial *hsp*60 gene sequences**. Neighbour-joining tree based on partial *hsp60 *gene sequences showing relationships among clinical isolate and members of the genus *Enterobacter *and *Buttiauxella*. Numbers at nodes indicate the level of bootstrap support (%) based on a neighbour-joining analysis of 1000 resampled datasets; only values above 50% are given. Bar, 0.1 or 0.01 substitutions per nucleotide.

**Figure 2:**
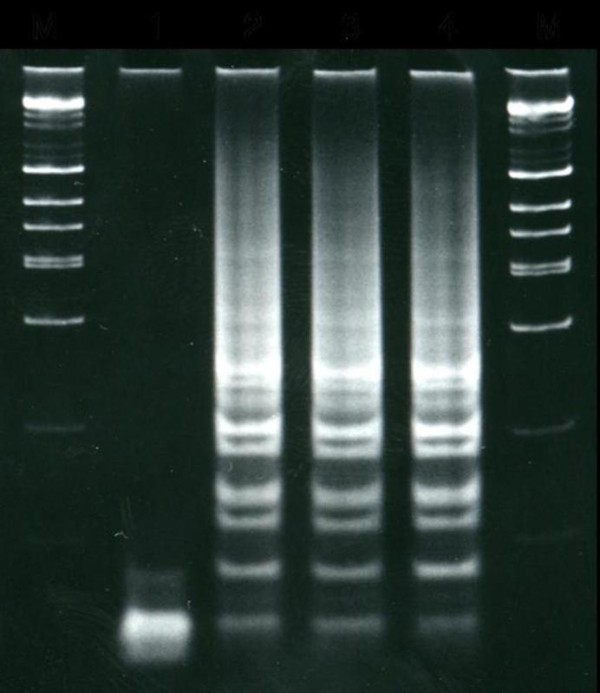
**IRS-PCR electrophoretic patterns of the *Enterobacter *species isolated**. Lane 1, negative control; lane 2, clinical isolate *Enterobacter nimipressuralis*,(Case 1); lane 3, Clinical isolate *Enterobacter nimipressuralis *(Case 2); lane 4, salined cotton isolate classified as *E. nimipressuralis*; M, DNA ladders.

The clinical isolates classified as *E. cloacae *and *E. amnigenus *were initially identified with a VITEK II automated system (bioMe´rieux, Marcy l'Etoile, France). Antimicrobial susceptibility determinations including the MICs were performed automatically with the VITEK II system. The Kirby-Bauer disk diffusion method was used next as a confirmatory tool. The isolates from both patients and the salined cotton container gave similar antibiogram and MIC results (Table 1). *Enterobacter *clinical isolates could not be identified in species level by 16S rRNA sequence due to the differences of sequence similarities among four species showing more than 99.0% identity with corresponding type strains. Previously, Tang YW *et. al*. also reported that the analysis of 16S rRNA gene is even though widely used for the bacterial identification, but it is poorly discriminatory for closely related members especially for the members of *Enterobacter *genus [1]. Hence, we performed sequence analysis of the protein encoding *hsp60 *gene and draw phylogenetic tree [2] (Figure 1). The *hsp60 *gene sequencing and phylogenetic analysis of amplification products revealed that clinical isolate shared maximum of 96.7% sequence similarity with *E. nimipressuralis *ATCC 9912*^T^*(GenBank accession number AJ567900). Hence, we assigned the clinical isolate as *E. nimipressuralis *species because it showed highest % similarity with this species, and clustered together in the same branch of the phylogenetic tree with *E. nimipressuralis *(Figure 1). Furthermore, we also performed infrequent-restriction-site PCR (IRS-PCR) [3] fingerprinting to confirm that the same bacterial strain had been isolated from the two patients and the salined cotton pad. Figure 2 shows that the three isolates yielded identical DNA banding patterns. Therefore all isolates were identified as *E. nimipressuralis *by hsp60 gene sequencing and IRS-PCR.

**Table 1:** Antibiotic susceptibility results showing MIC ((mg/ml) of the isolated *Enterobacter *species

Antimicrobial agent	Clinical isolate of Case 1	Clinical isolate of Case 2	Cotton isolate	CLSI Breakpoint ((g/ml)
ampicillin	16	16	16	8-32
Amoxacillin-clavulanic acid	< 4/< 4	< 4/< 4	< 4/< 4	8/4-32/16
Piperacillin-tazobactam	< 4/< 4	< 4/< 4	< 4/< 4	16/4-128/4
cefotaxime	< 1	< 1	< 1	8-64
ceftazidime	< 1	< 1	< 1	8-32
cefoxitin	8	8	8	8-32
cephalothin	> 64	> 64	> 64	8-32
imipenem	< 0.5	< 0.5	< 0.5	4-16
gentamicin	< 1	< 1	< 1	4-8
amikacin	< 2	< 2	< 2	16-32
netilmicin	< 1	< 1	< 1	12-32
trimethoprim-sulfamethoxazole	≤ 1/19	≤ 1/19	≤ 1/19	2/38-4/76
ciprofloxacin	< 0.25	< 0.25	< 0.25	1-4
ofloxacin	< 0.25	< 0.25	< 0.25	2-8
nitrofurantoin	16	32	16	32-128

*Enterobacter *species causes opportunistic infections, and could be primary human pathogens [4]. In the present study, although the clinical significance of the *E. nimipressuralis *remains unclear, the use of contaminated salined cotton at the venipuncture sites where blood was drawn seems the most likely explanation for the pseudobacteremia. Sterile salined cotton has been used instead of boric acid in the blood sampling room and Opthalmology Department since 2001. Boric acid cotton has not been used in our hospital since 2001 because of its weak antiseptic properties and significant toxicity [5]. Instead, sterile salined cotton is used before swabbing iodine to sample blood for culture. Sterile salined cotton should be supplied daily from the central supply department of our hospital: however, at the time of the outbreak, the salined cotton container had not been supplied adequately to the blood sample room.

When *E. amnigenus *or *E. nimipressuralis *is isolated from the patient who has no clinical signs of sepsis, a pseudobacteremia should be suspected, especially in cases where sensitivity testing and MIC results of bacterial cultures from two or more patients are nearly same, even though they appear to be different species. The microbiological culture monitoring system of sterile fluids by an infectious disease specialist may help to detect early other cases of pseudobacteremia. No further cases of *E. amnigenus *or *E. nimipressuralis *bacteremia have been occurred since we recommended using alcohol cotton for disinfection for sampling for blood culture and prohibited to use salined cotton for that purpose.

## Conclusion

Unusual microorganisms, such as *E. amnigenus *or *E. nimipressuralis*, isolated from blood of a patient with no clinical signs of sepsis should raise suspicion of pseudobacteremia. The microbiological culture monitoring system might be helpful to detect pseudobacteremia at early. The phoenotypic identification method Vitek II as well as the DNA sequence based typing methods with 16sRNA are not discriminatory enough to identify *E. cloacae *complex isolates, while sequencing a fragment of the *hsp*60 gene is more reliable for proper identification of *Enterobacter *species.

## Competing interests

The authors declare that they have no competing interests.

## Authors' contributions

DMK conceived of the study, participated in its design and coordination and drafted the manuscript, SJJ drafted the manuscript, took epidemiologic investigation, GPN, SHK and MSJ contributed in revision of the manuscript, SWK made a substantial contribution to draft the manuscript, and revised the draft all over the course of submission, WYK perform isolation and identification of *Enterobacter *spp. All authors read and approved the final manuscript.

## Pre-publication history

The pre-publication history for this paper can be accessed here:

http://www.biomedcentral.com/1471-2334/10/315/prepub
